# Prevalence of and risk factors for anal high-risk HPV among HIV-negative and HIV-positive MSM and transgender women in three countries at South-East Asia

**DOI:** 10.1097/MD.0000000000009898

**Published:** 2018-03-09

**Authors:** I Ketut Agus Somia, Nipat Teeratakulpisarn, Wifanto S. Jeo, Ilias A. Yee, Tippawan Pankam, Siriporn Nonenoy, Deondara Trachuntong, Pravit Mingkwanrungrueng, Made Dewi D. Sukmawati, Reshmie Ramautarsing, Hanny Nilasari, Nany Hairunisa, Iskandar Azwa, Evy Yunihastuti, Tuti P. Merati, Praphan Phanuphak, Joel Palefsky, Nittaya Phanuphak

**Affiliations:** a Division of Tropical and Infectious Disease, Department of Internal Medicine, Faculty of Medicine, Udayana University and Sanglah Hospital, Denpasar, Bali, Indonesia; b Prevention Department, Thai Red Cross AIDS Research Centre, Bangkok, Thailand; c Department of Surgery, School of Medicine Universitas Indonesia/HIV Integrated Clinic Cipto Mangunkusomo Hospital, Jakarta, Indonesia; d Clinical Investigation Centre, University Malaya Medical Centre, Kuala Lumpur, Malaysia; e Department of Dermatovenerology; f Department of Internal Medicine, School of Medicine Universitas Indonesia/HIV Integrated Clinic Cipto Mangunkusomo Hospital, Jakarta, Indonesia; g University of California San Francisco School of Medicine.

**Keywords:** Anal high-risk HPV, HIV-positive, Indonesia, Malaysia, MSM, Thailand, transgender women

## Abstract

This study aimed to assess the prevalence of and associated risk factors for anal high-risk human papillomavirus (hr-HPV) infection among men who have sex with men (MSM) and transgender women (TGW) in Indonesia, Thailand, and Malaysia.

This was baseline data from a prospective cohort study with clinic sites in Jakarta and Bali (Indonesia), Bangkok (Thailand), and Kuala Lumpur (Malaysia).

MSM and TGW aged 18 years and older from Indonesia, Thailand, and Malaysia were enrolled. Demographic and behavioral characteristics were assessed, and anal samples were collected for HPV genotyping. Multivariate logistic regression models were used to assess risk factors for anal hr-HPV overall and among HIV-positive participants.

A total of 392 participants were enrolled, and 48 were TGW. As many as 245 were HIV-positive, and 78.0% of the participants were on combination antiretroviral therapy (cART). Median CD4 count was 439 cells/mm^3^ and 68.2% had undetectable HIV-RNA. HIV-positive participants had significantly more hr-HPV compared to HIV-negative participants (76.6% vs 53.5%, *P* < .001). HPV-16 was the most common high-risk type (20%), whereas HPV-33, -39, and -58 were significantly more common among HIV-positive participants. HIV-positive participant significantly associated with anal hr-HPV infection compared with HIV-negative (OR: 2.87, 95% CI: 1.76–4.70, *P* ≤ .001), whereas among HIV-positive participants transgender identity had lower prevalence of hr-HPV infection (OR: 0.42, 95% CI: 0.19–0.91, *P* = .03).

High-risk HPV infection was very common among MSM and TGW in South-East Asia. Overall, HIV-infection, regardless of cART use and immune status, significantly increased the risk, while among HIV-positive participants transgender identity seemed to decrease the risk of anal hr-HPV.

## Abstract 2

Supplemental Digital Content is available in the text

## 1 Introduction

Men who have sex with men (MSM) are at an increased risk of anal cancer and the risk in HIV-positive MSM is 5 times higher than in HIV-negative MSM.^[1]^ The prevalence and incidence of the high-grade anal squamous intraepithelial lesion (HSIL), the putative precancerous lesion of anal cancer, continue to increase in the era of combination antiretroviral therapy (cART) among MSM with HIV infection,^[2–4]^ as does the incidence of anal cancer in this population.^[5,6]^ Anal infection with high-risk types of human papillomavirus (hr-HPV) is the most important risk factor for the development of HSIL and anal cancer.^[7–10]^ An estimated 80% of anal carcinoma is attributable to hr-HPV types 16 and 18.^[11]^

Transgender women (TGW) are at an increased risk of HIV infection, but data specific to this population are scarce, specifically due to a lack of inclusion of this population in national HIV surveillance systems.^[12]^ Data on HPV infection in this key population are even rarer.

Limited data are available regarding anal HPV infection and its associated diseases among MSM or TGW in South-East Asia. Preliminary data from MSM clinics in Indonesia and Thailand suggest a high rate of abnormal anal cytology among HIV-positive and HIV-negative MSM.^[13]^

This study aimed to assess the prevalence of and associated risk factors for anal hr-HPV infection among MSM and TGW in Indonesia, Thailand, and Malaysia.

## 2 Methods

This was baseline data from a prospective cohort study to assess anal hr-HPV infection and anal HSIL in MSM and TGW, with clinic sites in Jakarta and Bali (Indonesia), Bangkok (Thailand), and Kuala Lumpur (Malaysia). Here we report the prevalence of anal HPV infection. Between June 2013 and April 2015, MSM visiting 1 of the 4 clinics were invited to participate. Participants were eligible if they were Indonesian, Malaysian, or Thai citizens, at least 18 years of age, male at birth, and had a history of anal intercourse. They were not eligible for enrollment if they had prior treatment of anal cancer; anal cytology, high resolution anoscopy (HRA), or infrared coagulation within the previous 12 months; trichloroacetic acid/podophyllin application of the intra-anal area in the previous month; or evidence of active concurrent intra- or peri-anal bacterial or herpes simplex infection. The study was approved by all local institutional review boards. Informed consent has been obtained from all participants.

### 2.1 Demographic and risk behavior assessment

All participants were asked to complete questionnaires about demographic data and sexual and other behavioral risks. All questionnaires were in a Computer Assisted Self-Interview (CASI) format or in a self-administered paper questionnaire format.

### 2.2 Anal sample collection and HPV genotyping

An anal sample for HPV genotyping was collected by trained study physicians or nurses. A moistened Dacron or flocked swab was gently inserted 2 to 3 inches into the anal canal and removed with a 360-degree rotation to maximize cellular yield. The swab was shaken in liquid-based cytology fluid. The liquid-based cytology fluid was then tested for HPV using the Linear Array HPV Genotyping test (LA HPV GT; Roche Molecular Systems, Inc., Pleasanton, CA), which amplifies target DNA within the polymorphic L1 region of the HPV genome by polymerase chain reaction, then utilizes nucleic acid hybridization to independently identify 37 HPV DNA genotypes (6, 11, 16, 18, 26, 31, 33, 35, 39, 40, 42, 45, 51, 52, 53, 54, 55, 56, 58, 59, 61, 62, 64, 66, 67, 68, 69, 70, 71, 72, 73 [MM9], 81, 82 [MM4], 83 [MM7], 84 [MM8], IS39, and CP6108). These genotypes include the 13 high-risk genotypes: 16, 18, 31, 33, 35, 39, 45, 51, 52, 56, 58, 59, and 68. The primers for the human β-globin gene were used to ensure sample adequacy.

### 2.3 Other testing

All participants were tested for HIV and received pre- and post-test counseling, including risk reduction counseling. Screening for symptoms and signs of sexually transmitted diseases (STDs) was performed. Treatment for STDs was provided if needed, along with support for partner notification. Blood tests for the CD4 count and plasma HIV-RNA were performed for HIV-positive participants.

### 2.4 Staging HIV infection according CDC criteria

CDC classification A defined as asymptomatic, acute (primary) HIV infection or persistent generalized lymphadenopathy. Classification B defined as symptomatic conditions which occured in an HIV-infected adolescent or adult who meet at least one of the following criteria: attributed to HIV infection or indicate a defect in cell-mediated immunity; considered to have a clinical course or management that is complicated by HIV infection. While Classification C defined as having AIDS-indicator conditions.^[14]^

### 2.5 Statistical considerations

The sample size to determine the prevalence of anal hr-HPV infection among MSM and TGW was based on data from a cohort of Thai MSM followed at the Anonymous Clinic in Bangkok.^[15]^ In that cohort, the prevalence of hr-HPV infection was 57.5% in HIV-positive and 36.6% in HIV-negative MSM. A sample size of 430 participants in the present study would result in a 99% power to detect differences in the anal hr- HPV prevalence between HIV-positive and HIV-negative participants.

The demographic characteristics of study participants at each site, together with their baseline HIV-related characteristics, were assessed. The prevalence of anal HPV infection in HIV-positive and HIV-negative participants was calculated, together with 95% confidence intervals (CIs) around these point estimates. A series of logistic regression models were used to calculate the odds ratios (ORs) and 95% CIs for factors that were predictive of prevalent anal hr-HPV infections. Factors with a significance level of 0.2 or less in the univariate analysis were adjusted in a multivariate model by a backward stepwise selection method. In addition, a sub-analysis was performed among HIV-positive participants to model the effect of HIV-specific covariates, such as CD4 count, plasma HIV RNA level, and cART use. Data analyses were performed using STATA version 14 (StataCorp, College Station, TX).

## 3 Results

### 3.1 Demographic characteristics

The demographic characteristics are shown in Table 1 . A total of 392 participants (344 self-reported being MSM and 48 TGW) were enrolled in this study. As many as 245 participants (62.5%) were HIV-positive. HIV-positive participants were older and less likely to have a bachelor degree or higher compared to HIV-negative participants. Furthermore, they were less likely to use alcohol, more likely to have more lifetime sexual partners, and were more likely to use condoms during anal-insertive and oral-receptive intercourse compared to HIV-negative participants.

**Table 1:**
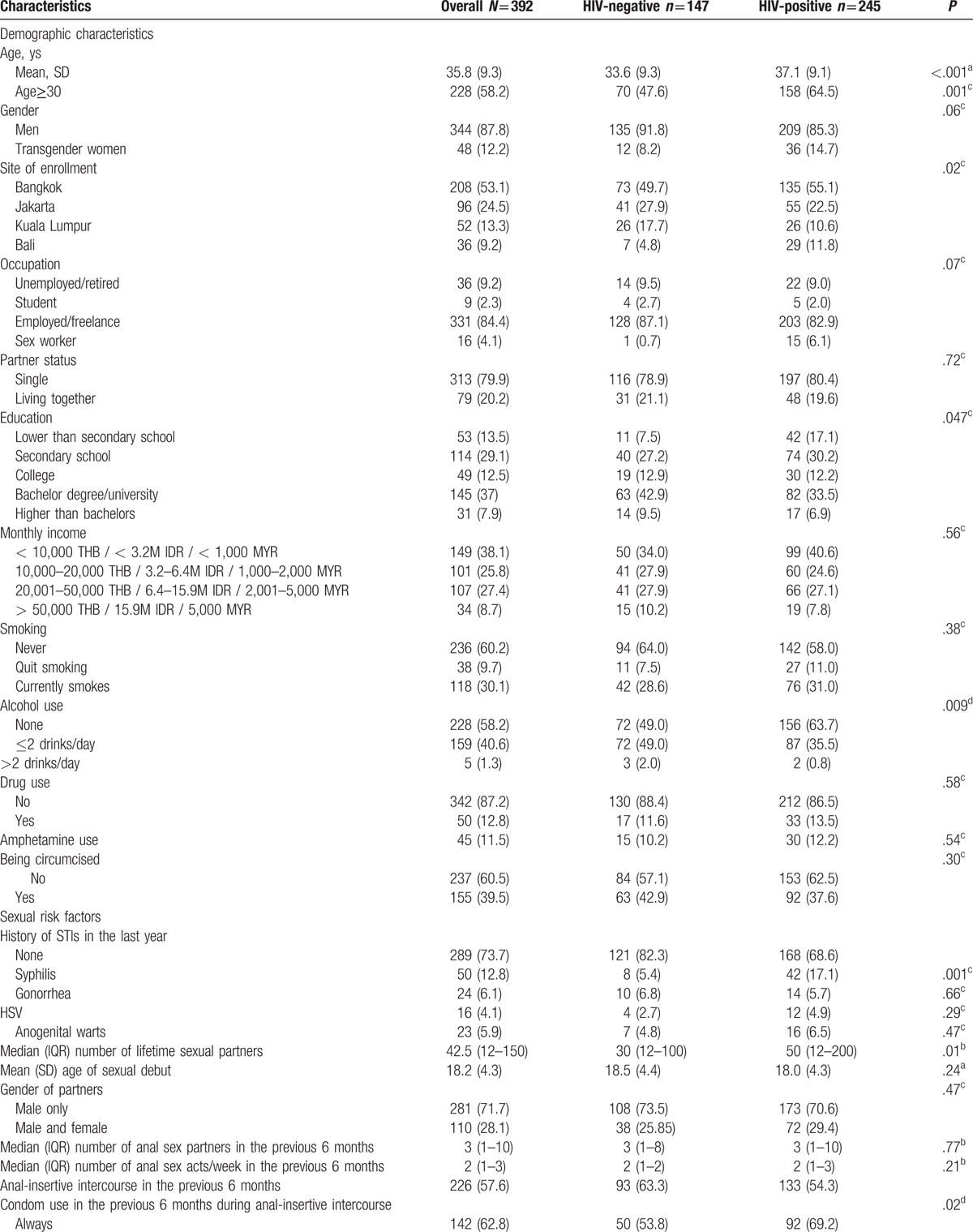
Baseline characteristics overall and stratified by HIV status.

**Table 1 (Continued):**
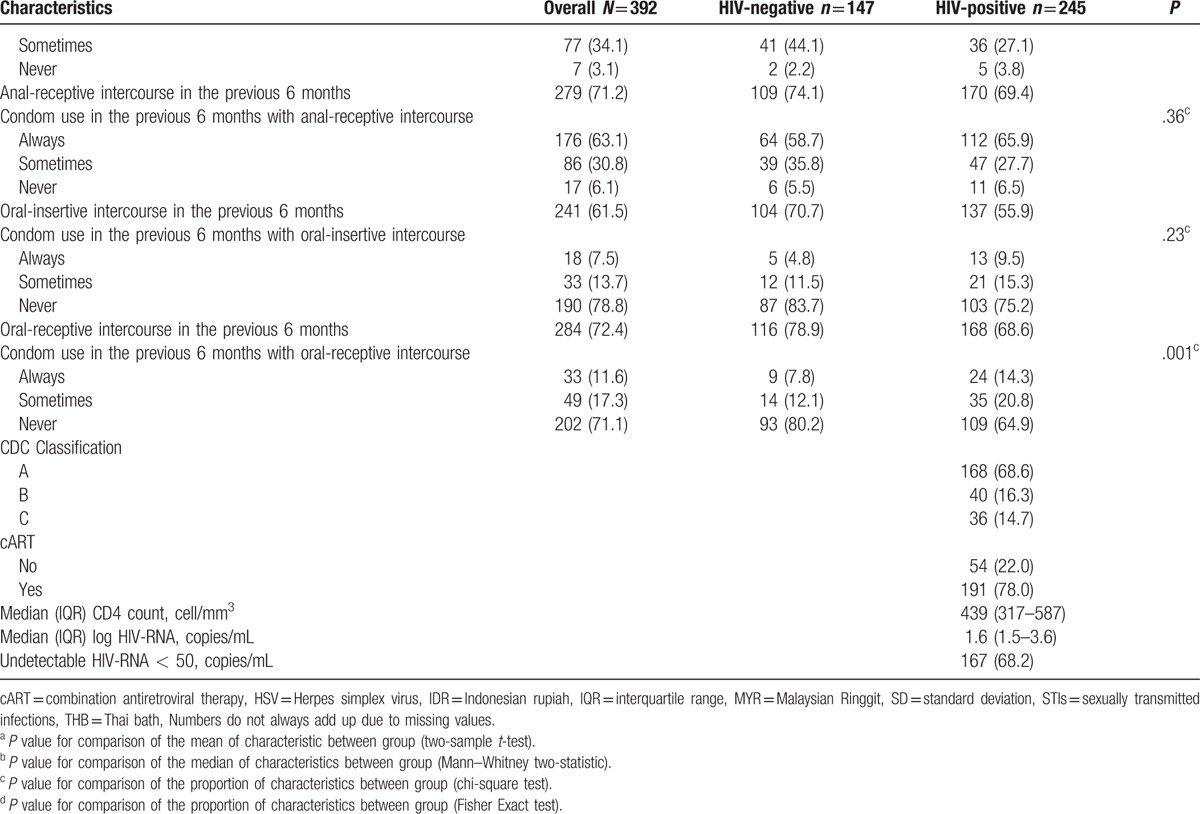
Baseline characteristics overall and stratified by HIV status.

Among the 245 HIV-positive participants, 209 self-identified as MSM and 36 as TGW (see Table 2 ). HIV-positive TGW were more likely to report sex work, to live together with their partner, to be circumcised, and to currently smoke than HIV-positive MSM. Furthermore, HIV-positive TGW reported a substantially higher number of lifetime sex partners, tended to be younger at their sexual debut, and more often had anal intercourse without a condom during the previous six months compared to HIV-positive MSM. The majority of HIV-positive participants were using cART and had undetectable plasma HIV-RNA. Participants from Bali were more likely to be HIV-positive (data not shown). Furthermore, none of the participants in Thailand or Malaysia self-identified as TGW. Based on partner status among the TGW (single and living together with partners), no difference in baseline characteristics was found between these two groups because of small samples and low power of test (see Table, Supplemental Digital Content 1, which presented baseline characteristics of TGW and stratified by partner status). HIV-positive participants from Bali had a lower CD4 count at baseline and were less likely to have undetectable HIV-RNA compared to other sites.

**Table 2:**
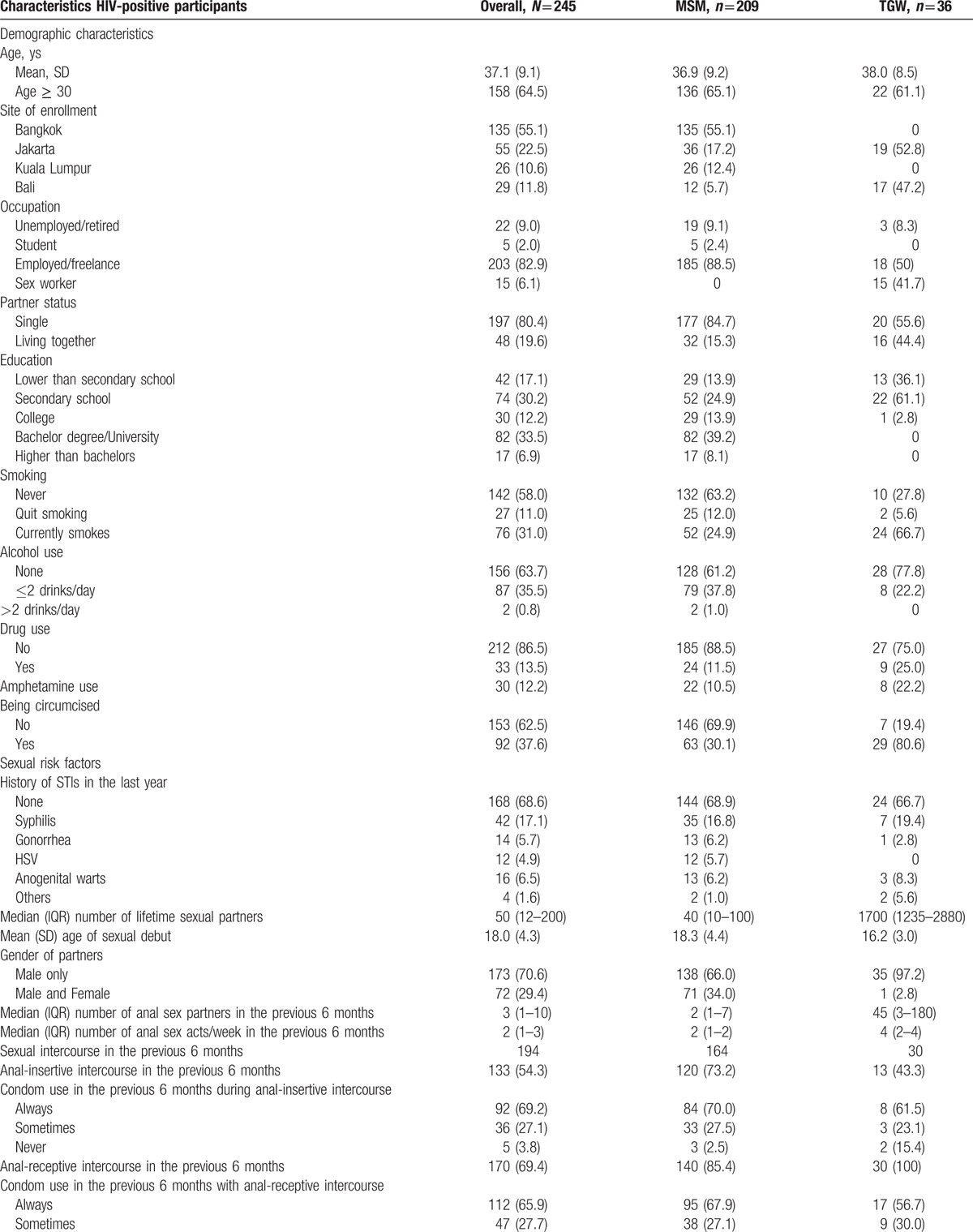
Baseline characteristics overall and stratified by gender.

**Table 2 (Continued):**
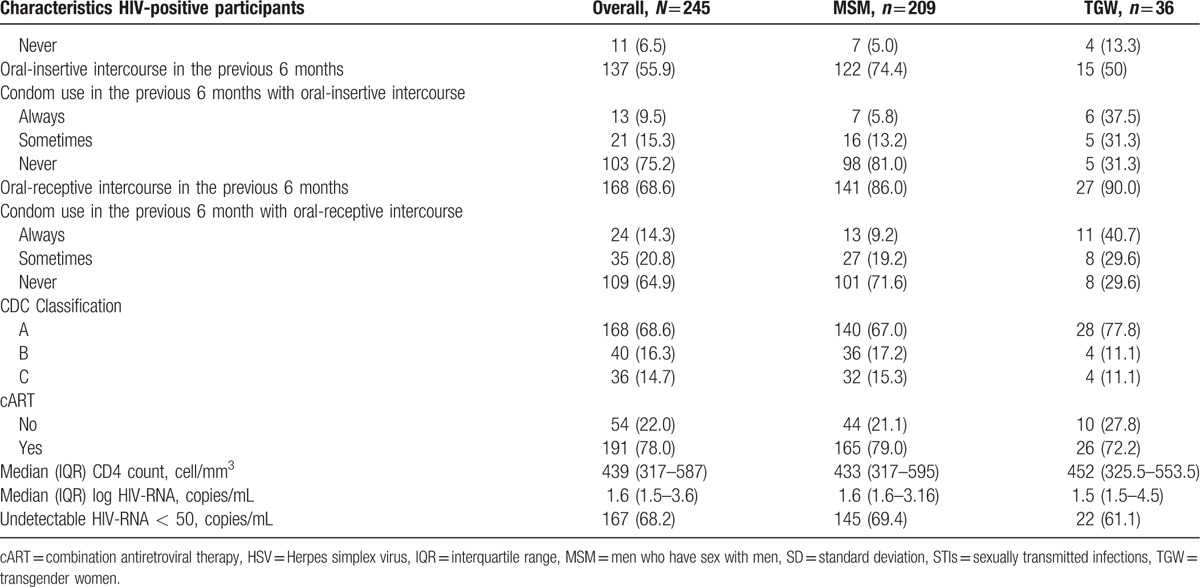
Baseline characteristics overall and stratified by gender.

### 3.2 Prevalence of any, low-risk, and high-risk anal HPV

Overall, any HPV was detected in 316 (80.6%) of participants. HIV-positive participants had a higher prevalence of any HPV (89.8%) compared to 65.3% in HIV-negative participants, *P* < .001; Table 3. They also had a higher prevalence of low-risk HPV infection (76.1% vs 43.7%, *P* < .001) and had more low-risk types present compared to HIV-negative participants.

**Table 3:**
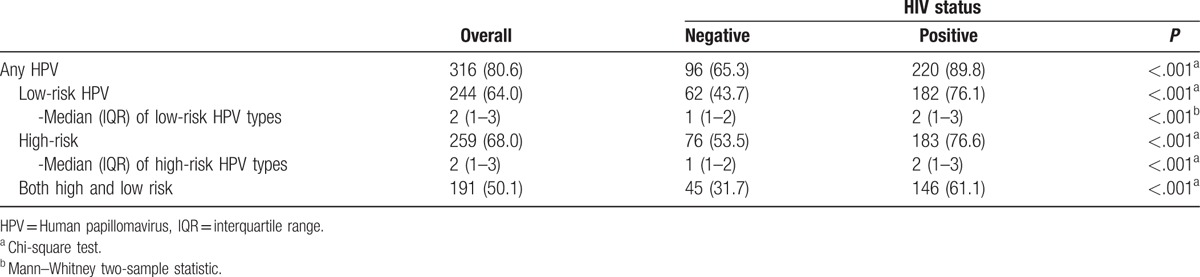
HPV prevalence among 381 participants (after excluding 11 participants with invalid results).

As much as 68% of all participants had anal hr-HPV. High-risk HPV was also more prevalent among HIV-positive MSM compared to HIV-negative MSM (76.6% vs 53.5%, *P* < .001), and the median number of high-risk types present was higher compared to HIV-negative MSM (2 vs 1 types, *P* < .001). Furthermore, 61% of HIV-positive participants had low-risk as well as high-risk HPV types present, compared to almost 32% of HIV-negative participants (*P* < .001). There was no difference in prevalence of any, low risk, or high-risk HPV infection among the four different clinical sites (data not shown).

### 3.3 Type-specific HPV prevalence

**Figure 1:**
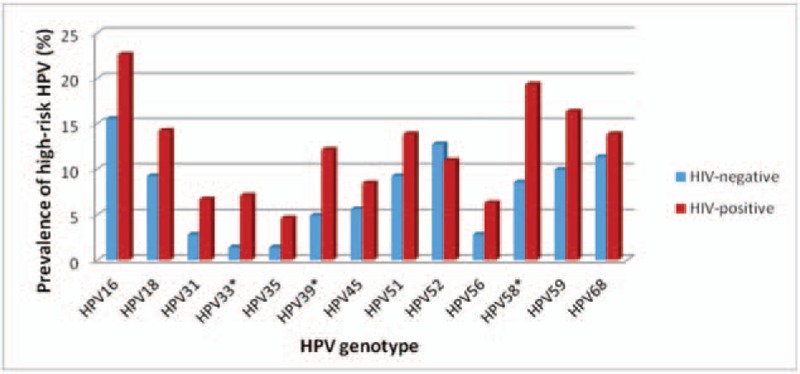
Anal high-risk HPV genotype distribution, by HIV status.

Fig. 1 shows the prevalence of anal hr-HPV types among HIV-positive and HIV-negative participants. The most common anal hr-HPV types found among HIV-positive participants were HPV16 (22.6%), HPV58 (19.3%), HPV59 (16.3%), HPV18 (14.2%), and HPV51 and HPV68 (both 13.8%). Among HIV-negative participants the most common high-risk HPV types were HPV16 (15.5%), HPV52 (12.7%), HPV68 (11.3%), HPV59 (9.9%), and HPV18 and HPV51 (both 9.2%). Among HIV-positive participants HPV33, HPV39, and HPV58 were significantly more prevalent than in HIV-negative participants (7.1% vs 1.4%, *P* = .01, 12.1% vs 4.9%, *P* = .02 and 19.3% vs 8.5%, *P* = .005, respectively) (see Table, Supplemental Digital Content 2, which showed prevalence of HPV type stratified by HIV status). The prevalence of HPV by site showed in Supplemental Digital Content 3 (see Table, Supplemental Digital Content 3, which showed prevalence of high and low-risk HPV types stratified by sites).

### 3.4 Risk factors associated with anal hr-HPV

Participants with an invalid HPV result were excluded from this analysis. A univariate analysis was performed to identify risk factors associated with anal hr-HPV infection among all participants. HIV status, partner status, and condom use for anal-receptive intercourse in the previous 6 months were significant at a level of 0.2 (Table 4). After adjustment in a multivariate model using a backward stepwise selection method, being HIV-positive was significantly associated with anal hr-HPV infection (OR: 2.87, 95% CI: 1.76–4.70, *P* < .001) compared with HIV-negative. Furthermore, living together was associated with a lower prevalence of having anal hr-HPV infection compared with being single (OR: 0.54, 95% CI: 0.30–0.95, *P* = .03). Age, history of STI, age at sexual debut, or other factors related to sexual behavior were not significantly associated.

**Table 4:**
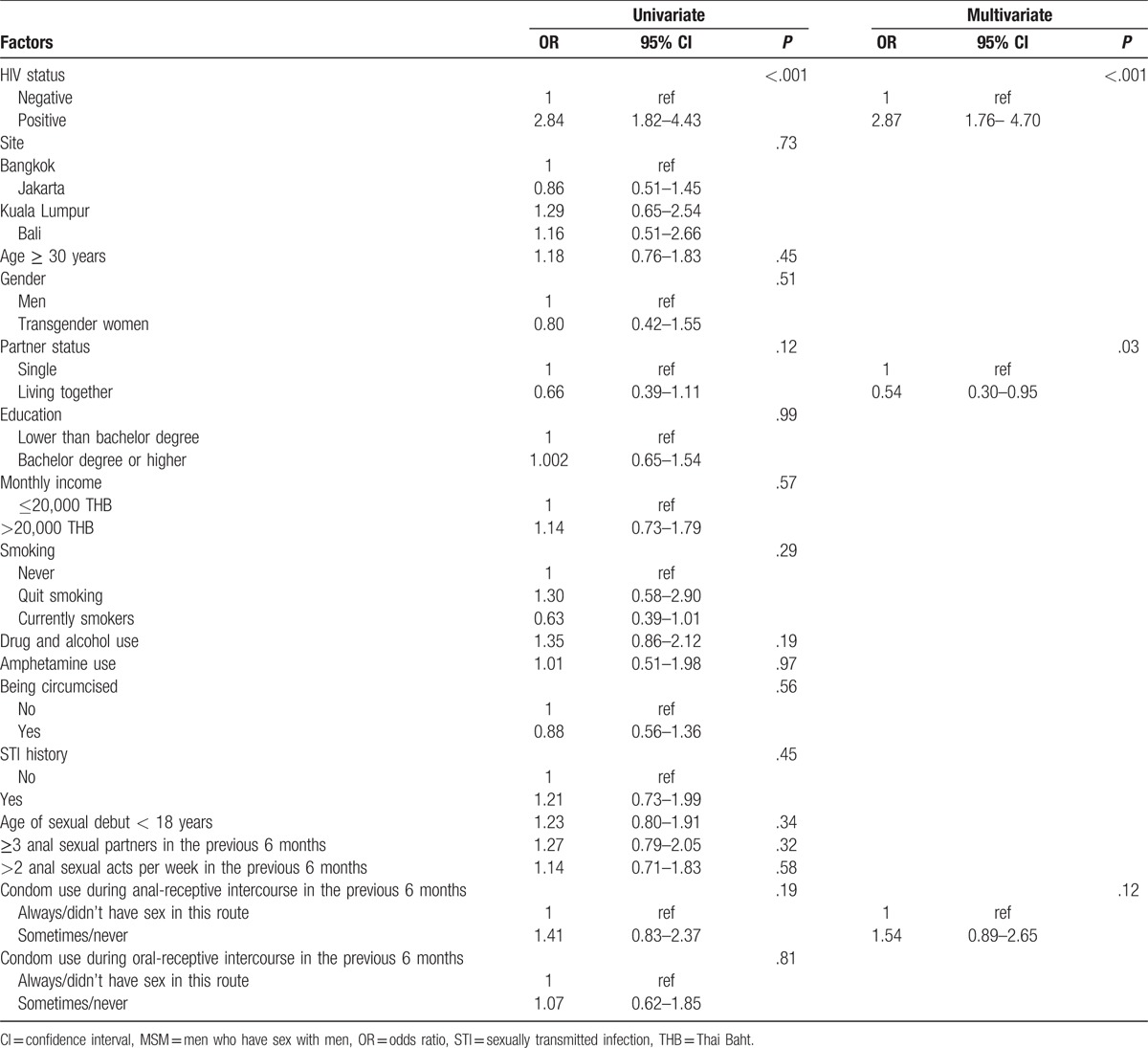
Logistic regression model of factors associated with prevalence of anal high-risk HPV types at baseline among HIV-positive and HIV-negative MSM.

We also examined risk factors for anal hr-HPV infection among HIV-positive participants (Table 5). Factors of significance in the univariate model were adjusted in a multivariate model using a backward stepwise selection method. TGW had a lower prevalence of anal hr-HPV (OR: 0.42, 95% CI: 0.19–0.91, *P* = .03). HIV-specific factors such as CD4 count, HIV-RNA, or use of cART were not significantly associated.

**Table 5:**
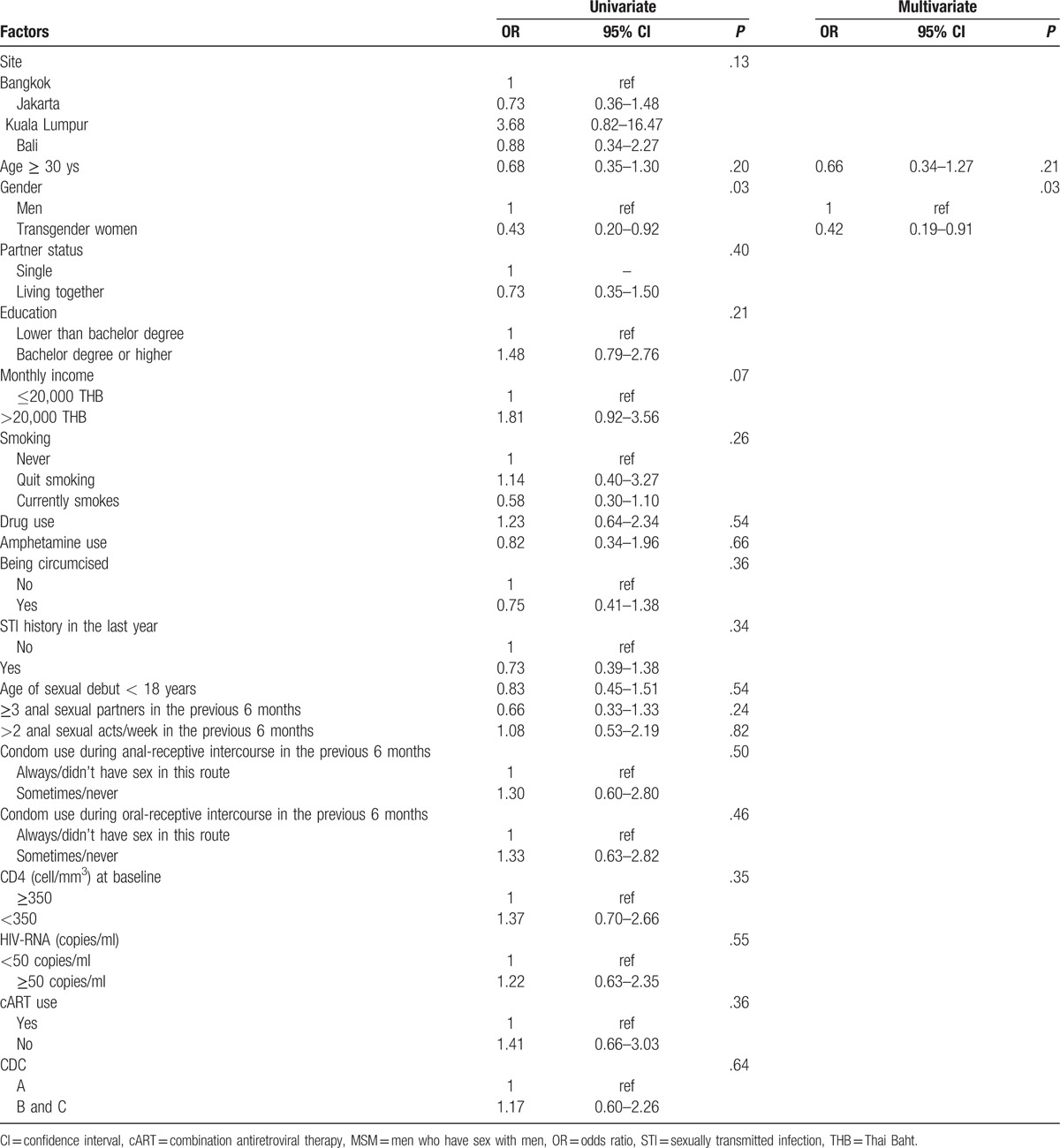
Logistic regression model of factors associated with prevalence of anal high-risk HPV types among HIV-positive MSM.

## 4 Discussion

In this study, we found that 80% of MSM and TGW in South-East Asia had any HPV in the anal canal, with a higher prevalence among HIV-positive participants (90% vs 65%, respectively) than HIV-negative participants. This prevalence is similar to the prevalence of any HPV we previously reported among HIV-positive and HIV-negative MSM in Bangkok.^[15]^ Furthermore, 68% of all participants had anal hr-HPV infection, again with a higher prevalence among HIV-positive participants than HIV-negative participants (76.6% vs 53.5%, respectively), which is higher than what we previously reported (57.7% and 36.6%, respectively),^[15]^ but lower than in North-Thailand (94% of HIV-positive MSM had anal hr-HPV, and 59% of HIV-negative MSM).^[16]^

Although HPV16 was the most common hr-HPV type found among HIV-positive as well as HIV-negative participants, high-risk types 33, 39, and 58 were significantly more common among HIV-positive participants than HIV-negative participants, and HIV-positive participants were more than twice as likely to have HPV 58 than HIV-negative participants. Furthermore, the prevalence of anal HPV16 among HIV-positive participants was 22.6%, which is lower than what has previously been reported in a meta-analysis looking at mostly North American studies (35.4%, 95% CI: 32.9–37.9).^[17]^ Our findings are consistent with several reports showing a different distribution of HPV serotypes. A recently published report from northern Thailand showed that among MSM and TGW the most common high-risk HPV types were 16 (27%), 58 (23%), 51 (18%), and 39 (14%).^[18]^ Among Indian HIV-infected MSM HPV16 was found in only 12% of the participants, compared to HPV35 which found in 20%, HPV33 in 8%, and HPV58 in 8% of the participants.^[19]^ Furthermore, among HIV-positive Nigerian MSM, HPV35 had the highest point prevalence (34.4%), followed by HPV58 (27.8%), HPV51 (26.7%), HPV18 (25.6%), HPV45 (25.6%), and HPV16 (23.3%).^[20]^ They also found that HIV infection was the strongest predictor of anal hr-HPV infection. The clinical relevance of this type-distribution found in anal swab samples is unclear. In 151 samples of anal cancer in the United Kingdom, 5 of whom were HIV-positive, HPV16 was found in 89% of anal cancers, HPV33 in 7%, and HPV58 in 5%.^[21]^ Worldwide, HPV16 was found in 80% of anal cancers, with HPV18 being the second most common type (3.6%), followed by HPV33 (2.7%), and HPV58 (1.8%).^[22]^ These findings seem to indicate that HPV33 and HPV58 infrequently cause anal cancer in the general population. However, the effect in an HIV-positive population might be different. It has been proposed that in women in Africa the immunodeficiency in HIV could alter the relative carcinogenicity of hr-HPV types, as a result of which a lower fraction of invasive cervical cancer is caused by HPV16.^[23]^

Among all MSM, HIV infection was an independent risk factor for anal hr-HPV infection. This has been reported consistently and is in line with a growing body of literature.^[15,20,24,25]^ Partner status was also associated with anal hr-HPV infection. Men who reported to be living together with their partner had a lower OR for having anal hr-HPV compared with being single. We know that sexual behavior is an important risk factor for anal HPV.^[26–28]^ Although we could not identify this in our study as an independent risk factor, living together with a stable partner might be a more reliable indicator of less risky sexual behavior than direct questions addressing this.

Interestingly, among HIV-infected participants, self-identifying as TGW significantly had a lower prevalence of anal hr-HPV compared to MSM (OR: 0.42, 95% CI: 0.19–0.91), even though HIV-positive TGW reported higher rates of risky behavior typically associated with hr-HPV infection, such as a younger age at sexual debut, a higher number of lifetime sexual partners, and unsafe anal intercourse. Furthermore, all 15 HIV-infected participants who reported sex work identified as TGW. Although none of the HIV-infected MSM reported sex work, this was not specifically addressed in the questionnaire and may, therefore, have been subject to response bias. HIV-associated factors, such as CD4 count, use of cART, or plasma HIV-RNA had no significant association, perhaps due to the high prevalence of anal hr-HPV infection in this group and the relatively small comparison group used for the regression model. These factors could explain why previous studies seem to contradict our findings; TGW appears to be at a higher risk of HPV infection and its related diseases. A study in north Thailand assessed anal hr-HPV infection in 200 participants, 85 of whom were TGW. There was no difference in anal cytological abnormalities between TWG and MSM.^[16]^ However, another report from the same group reported that compared to bisexual men, gay men had an OR of 4.2, and TGW had an even higher OR of 6.2 of having an anal hr-HPV infection.^[18]^ A study from India found that although not statistically significant, higher prevalence of anal dysplasia was evident in 24 TGW compared to 41 MSM (41.7% and 19.5%, respectively, *P* = .08).^[29]^ Furthermore, among 68 TGW in Peru, the majority of whom engaged in commercial sex work, 58.8% had anogenital (penile and anal combined) hr-HPV.^[30]^ It is unclear how our findings should be interpreted, especially given the observed higher rates of risky sexual behavior among TGW in our studies, such as more lifetime sexual partners, sex work, and a higher rate of unsafe anal intercourse. More research is needed to examine this further.

Infection with HPV types 6, 11, 16, and 18 can be prevented by the quadrivalent HPV vaccine Gardasil (Merck and Co, Whitehouse Station, NJ). The nonavalent HPV vaccine Gardasil 9 by the same manufacturer provides protection against five additional hr-HPV types: 31, 33, 45, 52, and 58. In addition to its approval for use in girls and women, both vaccines have been approved and recommended for use in boys and men. However, a few countries have adopted this recommendation into national vaccination programs for boys and men. A national vaccination program for HPV has not been implemented in Thailand and Indonesia, whereas the HPV vaccination program in Malaysia using Gardasil targets girls only.^[31]^ Our data indicate that there is a high burden of anal HPV infection among these 2 key populations and a broad hr-HPV type distribution among HIV-infected MSM and TGW. Implementation of routine HPV vaccination for boys in these countries using the nonavalent HPV vaccine will likely have a significant clinical impact.

Our study had an adequate sample size and enough power to detect a difference in anal hr-HPV prevalence between HIV-positive and HIV-negative participants. We also had some limitations. The number of TGW in the study was relatively small. Moreover, we did not collect information specifically about commercial sex work, which potentially could have been identified as a risk factor or further clarified our findings. Last, the logistic regression analyses were limited by the high rate of the outcome of interest in the study, which impacted the model.

Longitudinal information on incidence, clearance, and persistence of anal HPV infection and related diseases is needed to elucidate the disease burden further and to provide crucial epidemiological data to inform vaccination policies for these key populations. In addition, research specifically targeting TGW is needed to assess the risk of HPV and related diseases and design adequate prevention and treatment programs for this difficult-to-reach population.

## Acknowledgments

The authors would like to thank the ANSAP teams for their contributions.

## Supplementary Material

Supplemental Digital Content
