# Supplementary material for: Prevalence of and risk factors for anal high-risk HPV among HIV-negative and HIV-positive MSM and transgender women in three countries at South-East Asia
Source: Medicine (Baltimore). 2018 Mar 9;97(10):e9898. doi: 10.1097/MD.0000000000009898 (PMC5882413; doi:10.1097/MD.0000000000009898)
Supplement: Supplemental Digital Content [file medi-97-e9898-s001.pdf]

Supplemental Digital Content 1

Baseline characteristics of TGW and stratified by partner status

Characteristics	TGW (n = 36)	Single (n=20)	Living together with partners (n=16)	P-value†
**Occupation**				0.19
Unemployed/home duties /retired/street singer	3(8.3)	0(0)	3(18.8)	
Employed/freelance	18(50)	11(55)	7(43.8)	
Sex worker	15(41.7)	9(45)	6(37.5)	
**STI history**				
None	24(66.7)	14(70)	10(62.5)	
Syphilis	7(19.4)	3(15)	4(25)	0.68
Gonorrhea	1(2.8)	0(0)	1(6.3)	0.44
Herpes simplex (HSV) infection	0(0.0)	0(0)	0(0)	-
Non-specific urethritis	0(0.0)	0(0)	0(0)	-
Anogenital warts, location	3(8.3)	3(15)	0(0)	0.24
Other	2(5.6)	1(5)	1(6.3)	>0.99
**lifetime sexual behavior**				
**Number of lifetime sexual partners**				
Median(IQR)	1700(1235-2880)	1650(750-2000)	1700(1500-6785)	0.29

Characteristics	TGW (n = 36)	Single (n=20)	Living together with partners (n=16)	P-value†
**Age of sexual debut (years)**				
Median(IQR)	15.5(14.0-18.0)	14.5(12.5-17.5)	17(15-19)	0.15
**Recent sexual behavior**				
**In the past 6 months, number of anal sexual partners**				
Median(IQR)	45(3-180)	50(3.5-150)	42.5(3-180)	0.29
**In the past 6 months, frequency of anal sexual acts per week**				
Median(IQR)	4(2-4)	4(2-4)	4(2-4)	0.32
**In the past 6 months, any sexual partners with known HIV-positive status**				>0.99
Yes	2(5.6)	1(5)	1(6.3)	
No	4(11.1)	2(10)	2(12.5)	
Not sure	24(66.7)	13(65)	11(68.8)	
Missing	6(16.7)	4(20)	2(12.5)	
**Anal insertive, Number who had sex activity in this route**	**13**	**10**	**3**	
Condom used in the past 6 month,				0.71

Characteristics	TGW (n = 36)	Single (n=20)	Living together with partners (n=16)	P-value†
Always	8(61.5)	5(50)	3(100)	
Sometimes	3(23.1)	3(30)	0(0)	
Never	2(15.4)	2(20)	0(0)	
**Anal receptive, Number who had sex activity in this route**	**30**	**16**	**14**	
Condom used in the past 6 month,				0.40
Always	17(56.7)	10(62.5)	7(50)	
Sometimes	9(30.0)	3(18.8)	6(42.9)	
Never	4(13.3)	3(18.8)	1(7.1)	
**Oral insertive, Number who had sex activity in this route**	**15**	**11**	**4**	
Condom used in the past 6 month,				>0.99
Always	6(37.5)	4(33.3)	2(50)	
Sometimes	5(31.3)	4(33.3)	1(25)	
Never	5(31.3)	4(33.3)	1(25)	
**Oral receptive, Number who had sex activity in this route**	**27**	**16**	**11**	
Condom used in the past 6 month,				0.38
Always	11(40.7)	7(43.8)	4(36.4)	
Sometimes	8(29.6)	3(18.8)	5(45.5)	

Characteristics	TGW (n = 36)	Single (n=20)	Living together with partners (n=16)	P-value†
Never	8(29.6)	6(37.5)	2(18.2)	

† Fisher' s Exact test for comparison of proportion of characteristics between groups
Mann-Whitney two-statistic for comparison of median of characteristics between groups

Supplemental Digital Content 2

Prevalence of high and low-risk HPV types stratified by HIV status

(excluded 11 participants who have HPV result as invalided, N = 381)

HPV Type	Overall n = 381	HIV status		
		HIV negative n = 142	HIV positive n = 239	*p*-value
**High risk**				
**HPV16**	76 (20)	22 (15.5)	54 (22.6)	0.09[a]
**HPV18**	47 (12.3)	13 (9.2)	34 (14.2)	0.15[a]
**HPV31**	20 (5.3)	4 (2.8)	16 (6.7)	0.10[a]
**HPV33**	19 (5)	2 (1.4)	17 (7.1)	**0.01[a]**
**HPV35**	13 (3.4)	2 (1.4)	11 (4.6)	0.14[b]
**HPV39**	36 (9.5)	7 (4.9)	29 (12.1)	**0.02[a]**
**HPV45**	28 (7.4)	8 (5.6)	20 (8.4)	0.32[a]
**HPV51**	46 (12.1)	13 (9.2)	33 (13.8)	0.18[a]
**HPV52**	44 (11.6)	18 (12.7)	26 (10.9)	0.60[a]
**HPV56**	19 (5)	4 (2.8)	15 (6.3)	0.13[a]
**HPV58**	58 (15.2)	12 (8.5)	46 (19.3)	**0.005[a]**
**HPV59**	53 (13.9)	14 (9.9)	39 (16.3)	0.08[a]
**HPV68**	49 (12.9)	16 (11.3)	33 (13.8)	0.47[a]
**Any high risk HPV**	259 (68)	76 (53.5)	183 (76.6)	**< 0.001[a]**
**Low risk**				
**HPV6**	48 (12.6)	12 (8.5)	36 (15.1)	0.06[a]
**HPV11**	64 (16.8)	16 (11.3)	48 (20.1)	**0.03[a]**
**HPV26**	17 (4.5)	6 (4.2)	11 (4.6)	0.86[a]
**HPV40**	12 (3.2)	0 (0.0)	12 (5.0)	**0.005[b]**
**HPV42**	2 (6)	10 (7.0)	13 (5.4)	0.52[a]
**HPV53**	31 (8.1)	9 (6.3)	22 (9.2)	0.32[a]
**HPV54**	20 (5.3)	5 (3.5)	15 (6.3)	0.24[a]
**HPV55**	26 (6.8)	6 (4.2)	20 (8.4)	0.12[a]
**HPV61**	19 (5)	4 (2.8)	15 (6.3)	0.13[a]
**HPV62**	37 (9.7)	6 (4.2)	31 (13)	**0.005[a]**
**HPV64**	1 (0.3)	0 (0.0)	1 (0.4)	-
**HPV66**	26 (6.8)	4 (2.8)	22 (9.2)	**0.02[a]**
**HPV67**	6 (1.6)	1 (0.7)	5 (2.1	0.42[b]
**HPV69**	14 (3.7)	1 (0.7)	13 (5.4	**0.02[a]**
**HPV70**	31 (8.1)	8 (5.6)	23 (9.6	0.17[a]
**HPV71**	7 (1.8)	0 (0)	7 (2.9	**0.049[b]**

**HPV72**	17 (4.5)	2 (1.4)	15 (6.3	**0.03**[a]
**HPV73**	22 (5.8)	5 (3.5)	17 (7.1	0.15[a]
**HPV81**	22 (5.8)	4 (2.8)	18 (7.5	0.056[a]
**HPV82**	13 (3.4)	2 (1.4)	11 (4.6	0.14[b]
**HPV83**	11 (2.9)	2 (1.4)	9 (3.8	0.22[b]
**HPV84**	29 (7.6)	5 (3.2)	24 (10.0	**0.02**[a]
**CP6108**	22 (5.8)	4 (2.8)	18 (7.5	0.056[a]
**IS39**	9 (2.4)	2 (1.4)	7 (2.9	0.49[b]
**Any low risk HPV**	244 (64)	62 (43.7)	182 (76.2)	**< 0.001**[a]

a = Chi-square test

b = Fisher's Exact test

Supplemental Digital Content 3

Prevalence of high and low-risk HPV types Stratified by sites

(excluded 11 participants who have HPV result as invalided, N = 381)

**HPV Type**	**Sites**				
	**Thailand n = 205**	**Jakarta n = 93**	**Kuala Lumpur n = 52**	**Bali n = 31**	***p*-value**
**High risk**					
**HPV16**	38(18.5)	16(17.2)	17(32.7)	5(16.1)	0.10[a]
**HPV18**	28(13.7)	8(8.6)	6(11.5)	5(16.1)	0.58[a]
**HPV31**	10(4.9)	3(3.2)	7(13.5)	0(0.0)	**0.04**[b]
**HPV33**	13(6.3)	1(1.1)	3(5.8)	2(6.5)	0.16[b]
**HPV35**	6(2.9)	2(2.2)	5(9.6)	0(0.0)	0.10[b]
**HPV39**	15(7.3)	10(10.8)	7(13.5)	4(12.9)	0.35[b]
**HPV45**	14(6.8)	7(7.5)	5(9.6)	2(6.5)	0.88[b]
**HPV51**	18(8.8)	13(14.0)	10(19.2)	5(16.1)	0.14[a]
**HPV52**	20(9.8)	13(14.0)	5(9.6)	6(19.4)	0.36[a]
**HPV56**	8(3.9)	4(4.3)	3(5.8)	4(12.9)	0.19[b]
**HPV58**	35(17.1)	10(10.8)	7(13.5)	6(19.4)	0.47[a]
**HPV59**	28(13.7)	15(16.1)	7(13.5)	3(9.7)	0.83[a]
**HPV68**	22(10.7)	14(15.1)	7(13.5)	6(19.4)	0.49[a]
**Any high risk HPV**	139(67.8)	60(64.5)	38(73.1)	22(71)	0.74[a]
**Low risk**					
**HPV6**	25(25.8)	9(11.7)	11(6.6)	3(3.9)	0.22[a]
**HPV11**	31(15.1)	19(20.4)	10(19.2)	4(12.9)	0.60[a]
**HPV26**	8(3.9)	6(6.5)	2(3.9)	1(3.2)	0.80[b]
**HPV40**	8(3.9)	3(3.2)	0(0.0)	1(3.2)	0.61[b]
**HPV42**	10(4.9)	9(9.7)	4(7.7)	0(0.0)	0.18[b]
**HPV53**	17(8.3)	7(7.5)	3(5.8)	4(12.9)	0.72[b]
**HPV54**	9(4.4)	4(4.3)	3(5.8)	4(12.9)	0.26[b]
**HPV55**	12(5.9)	7(7.5)	4(7.7)	3(9.7)	0.73[b]
**HPV61**	6(2.9)	8(8.6)	2(3.9)	3(9.7)	0.08[b]
**HPV62**	17(8.3)	14(15.1)	2(3.9)	4(12.9)	0.11[b]
**HPV64**	1(0.5)	0(0.0)	0(0/0)	0(0.0)	-
**HPV66**	18(8.8)	5(5.4)	3(5.8)	0(0.0)	0.31[b]
**HPV67**	2(1.0)	1(1.1)	2(3.9)	1(3.2)	0.21[b]
**HPV69**	6(2.9)	4(4.3)	3(5.8)	1(3.2)	0.64[b]

**HPV70**	17(8.3)	7(7.5)	2(3.9)	5(16.1)	0.29[b]
**HPV71**	5(2.4)	1(1.1)	0(0.0)	1(3.2)	0.50[b]
**HPV72**	9(4.4)	2(2.2)	2(3.9)	4(12.9)	0.12[b]
**HPV73**	15(7.3)	3(3.2)	3(5.8)	1(3.2)	0.60[b]
**HPV81**	11(5.4)	4(4.3)	5(9.6)	2(6.5)	0.55[b]
**HPV82**	4(2.0)	2(2.2)	4(7.7)	3(9.7)	**0.03[b]**
**HPV83**	4(2.0)	4(4.3)	1(1.9)	2(6.5)	0.28[b]
**HPV84**	18(8.8)	4(4.3)	5(9.6)	2(6.5)	0.51[b]
**CP6108**	12(5.9)	3(3.2)	6(11.5)	1(3.2)	0.23[b]
**IS39**	2(1.0)	5(5.4)	1(1.9)	1(3.2)	0.09[b]
**Any low risk HPV**	124(60.5)	60(64.5)	34(65.4)	26(83.9)	0.09[a]

a = Chi-square test

b = Fisher's Exact test
